# Quantitative proteomics reveal three potential biomarkers for risk assessment of acute myocardial infarction

**DOI:** 10.1080/21655979.2022.2037365

**Published:** 2022-02-14

**Authors:** Yao Xie, Hongzhou Zhang, Tieqiu Huang

**Affiliations:** Department of Cardiovascular Medicine, The Second Affiliated Hospital of Nanchang University, Nanchang, China

**Keywords:** Proteomics, acute myocardial infarction, biomarker, exosome

## Abstract

Acute myocardial infarction (AMI) is the one of the main cause of death worldwide. Exosomes carry important information about intercellular communication and could be diagnostic marker for many diseases. Here, we aimed to find potential key proteins for the early diagnosis of AMI. A label free proteomics strategy was used to identify the differentially expressed proteins (DEPs) of AMI patients’ plasma exosome. By bioinformatics analysis and enzyme-linked immunosorbent assay to validate the candidate proteins. Compared to healthy control plasma exosome, we totally identified 72 differentially expressed proteins (DEPs) in AMI patients. Also, we found that complement and coagulation cascades was activated by KEGG analysis and GSEA. PLG, C8B and F2 were selected as candidate molecules for further study, and then validated another 40 plasma samples using enzyme-linked immunosorbent assay. Finally, we found that the expression levels of these three proteins (PLG, C8B and F2) were significantly higher than those of healthy controls (P < 0.05). ROC analysis revealed that PLG, C8B and F2 had potential value for AMI early diagnosis. In conclusion, our study identified three potential biomarkers for AMI diagnosis. But there remains a need to further study the mechanism of the biomarkers.

## GRAPHICAL ABSTRACT

**Figure:**
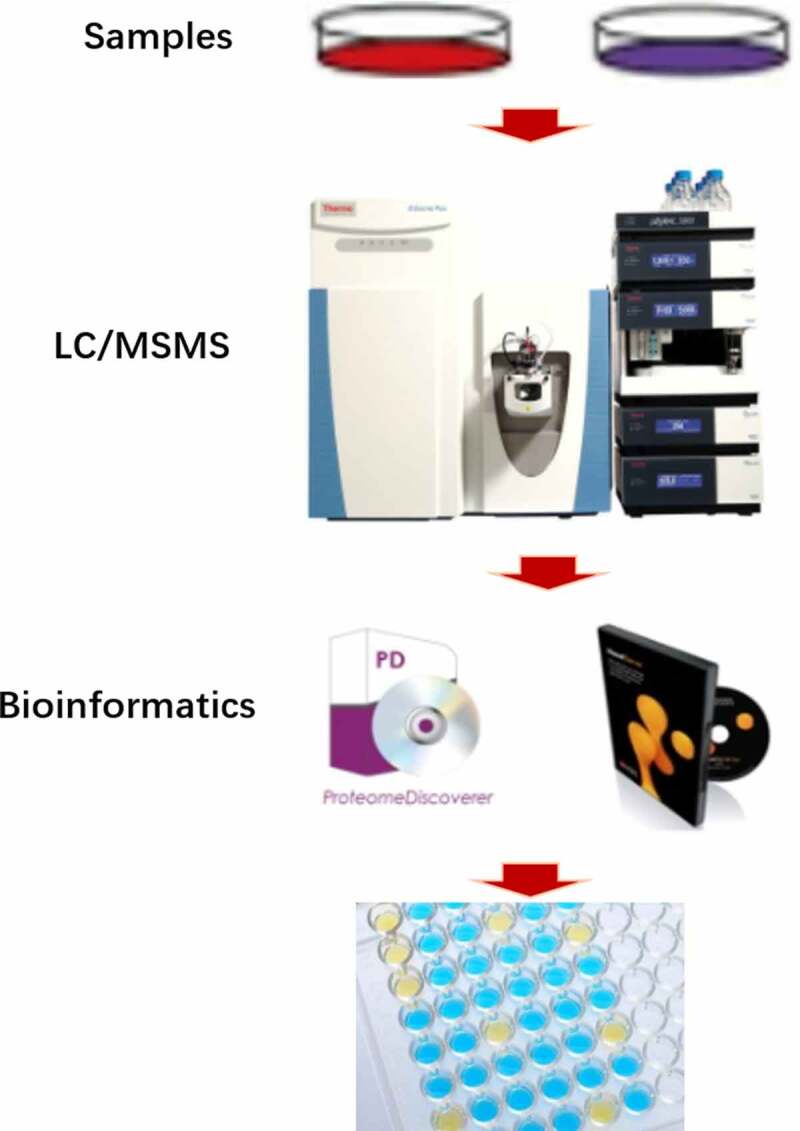

## 1. Introduction

The incidence of acute myocardial infarction (AMI) is increasing year by year, and it is also one of the main causes of death worldwide [1,2]. Myocardial cell death and subsequent excessive inflammation are the major causes of heart damage in AMI, which can eventually lead to heart failure and sudden death [3]. Thus, identifying key biomarkers during the acute myocardial infarction is of great importance for developing potential novel therapeutic strategies.

Exosomes are particles originated from cellular endosomes, ranging in size from 40 to 100 nm. It contains a variety of bioactive components [4]. Studies have shown that exosomes from blood may reflect the pathological state of the derived cells from which they originate, and thus can be used as diagnostic biomarkers for cardiovascular disease [5,6]. In recent years, many studies have shown that exosomes are important carriers for extracellular communication and have systematic effects on biological physiology [7,8]. Cardiac exosomes play a role in a variety of cardiac pathologic conditions, including AMI. Several studies showed injured cardiomyocytes could release exosomes, which are enriched with cardiac-specific miRNAs [9]. These evidences suggest some potential diagnostic biomarkers for the early diagnosis of AMI [10]. However, the landscape of protein profile of exosome in AMI is rarely reported.

In the study, we aim to identify novel candidate biomarkers in AMI. As exosomes can promote information communication between cells by exchanging proteins, we hypothesized that proteins specific to AMI patients could be identified by serum exosomes. Here, we performed a label-free proteomics detection to discover differentially expressed proteins from plasma exosome of patients and healthy subjects. Also, ELISAs were used to validate our results.

## 2. Materials and methods

### 2.1. Clinical samples

For this study, six AMI patients (aged 41–78 years; 4 people ≥60 years and 2 people<60) and six healthy controls (aged 43–81 years; 4 people ≥60 years and 2 people<60) for protomics study, and another 20 AMI (aged 41–78 years;12 people ≥60 years and 8 people<60) and 20 healthy controls (aged 43–81 years;13 people ≥60 years and 7 people<60) for validation were recruited from the Second Affiliated Hospital of Nanchang University. Patients were diagnosed according to the guidelines of the American Heart Association/American College of Cardiology Foundation [11–13]. AMI patients were diagnosed using the changes in myocardial necrotic proteins and met one of the following criteria: (I) showing clear ischemic symptoms; (II) showing dynamic electrocardiogram (ECG) changes with pathological Q wave; (III) showing new ST-T changes or onset of left bundle branch block on ECG; or (IV) showing segmental wall motion disorder on imaging, or with loss of viable myocardium [11,12]. The patients with hepatitis B, tuberculosis, diabetes, AIDS, cancer, and immunosuppressive drug users were excluded. Fasting blood samples were collected in the morning from all donators. 5.0 mL peripheral blood were collected and centrifuged at 15,000 g at 4°C for 5 min to separate the plasma. Then, all the plasma from the cases was collected and preserved in liquid nitrogen. The research ethics committee board of the Second Affiliated Hospital of Nanchang University approved this study (No.2019057). All the patients participant included in the study submitted written informed consent forms.

### 2.2. *Exosome isolation* [14]

Exosomes were extracted from the plasma of AMI patients and healthy individuals using an ExoQuick exosome precipitation solution kit (System Biosciences, Mountain View, CA, USA) according to the manufacturer’s instructions. Briefly, 300 μL of plasma were mixed with 80 μL of the solution and then incubated for 1 hour at 4°C. Subsequently, the mixture was centrifuged at 3,000*g* for 30 min at 25°C, and the precipitation obtained after the removal of supernatant was resuspended in 250 μL of 1X PBS solution. The isolated exosomes were stored at −80°C.

### 2.3. *Transmission electron microscopy* [15]

The fifteen microliters of isolated exosomes were moved onto formvar carbon coated copper mesh, allowed to adsorb for 10 min before excess fluid was drained. The adsorbed exosomes were then negatively stained with 3% (w/v) phosphortungstate acid (pH 6.8) for 5 min. The exosomes were then air-dried under an electric incandescent lamp and analyzed by transmission electron microscopy (Fei Tecnai 12, Philips) at bar = 100 nm.

### 2.4. *Size and concentration analyses of exosomes* [15]

In addition, the isolated exosomes were diluted with PBS and analyzed using a NanoSight LM10 Instrument (NanoSight, Malvern, UK) following the manufacturer’s protocol. Nanoparticle Tracking Analysis 2.0 (NTA 2.0) software was used to analyze the size and concentration of exosomes.

### 2.5. *Protein preparation* [16]

The exosomes were ground in liquid nitrogen, in liquid nitrogen and then lysed in buffers (1% SDS, 7 M urea, 1X protease inhibitor cocktail (Roche)) was added to the sample and then vortexed and pulverized 3 times for 10 minutes. The ice was cracked for 30 min and centrifuged at 15,000 rpm for 15 min at 4°C. Then, the supernatant was collected and the protein concentration of the supernatant was determined by the Bradford protein assay (Thermo Fisher, USA).

### 2.6. *Western blot* [17]

20ug protein was taken from each sample for SDS-PAGE electrophoresis, proteins were transferred to PVDF membranes. The membranes were blocked overnight at 4°C and 5% non‐fat dry milk in TBS‐T buffer (15 mM Tris, pH7.8, 100 mM NaCl, 0.5% Tween‐20), followed by 3 h of incubation with the primary antibody (1:1,500–1:2,500 dilution) in TBS‐T buffer containing 5% non‐fat dry milk at 25°C. After washing with TBS-T buffer for three times, horseradish peroxidase labeled goat anti-mouse IgG, goat anti-rabbit IgG or rabbit anti-goat IgG were used as secondary antibodies (1:2000 dilution) and incubated for 1 h at room temperature. The reaction is then washed 3 times in TBS‐T buffer and visualized by the ECL detection system. All of the Western blot assays were repeated at least 3 times.

### 2.7. *Label-free quantification* [18]

The protein peptides were performed on a Q-Extractive HF mass spectrometer (Thermo Scientific) coupled with an Ultimate 3000 system (Thermo Scientific). The MS raw data was searched by Mascot 2.0 (Matrix Science, London, UK) using Uniprot database (https://www.uniprot.org/, human, October 2019). Relative abundance of peptide signatures (precursor peak area) was detected in multiple samples. The features of the peptides in different samples were aligned reliably with each other using an efficient retention time alignment algorithm. The total ionic current (TIC) of the sample was normalized, and the normalized abundance was calculated by dividing the original abundance by the normalized factor.

### 2.8. *Enzyme-linked immunosorbent assays (ELISAs)* [19]

The plasma of another 20 AMI (aged 41–78 years) and 20 healthy controls (aged 43–81 years) were used to validation, and the plasma was collected and centrifuged at 3000 g for 15 minutes. The concentrations of PLG, C8B and F2 were determined by ELISA using a human PLG ELISA kit (#ab100629, abcam), human F2 ELISA kit (#ab270210, abcam), and human C8B ELISA kit (#SEB306Hu, Cloud-clone Corp). The ELISAs were performed according to each manufacturer’s instructions.

### 2.9. Statistical analysis

The AMI group was compared with the normal group (NC) by unpaired student t test. Welch’s ANOVA test was utilized to analyze the difference between the AMI and healthy control groups. P-value <0.05 and |fold change|> 1.3 were used for DEPs screening. Receiver operating characteristic (ROC) curve was used to analyze the diagnosis value of the candidate DEPs, including the area under the curve (AUC), sensitivity, and specificity.

## 3. Results

This study aims to reveal novel candidate biomarkers in AMI. Exosomes were isolated from the plasma of healthy controls and AMI patients. Then, using the label-free proteome strategy to identify the DEPs between the two groups. Bioinformatic analyses showed the complement and coagulation cascades pathway were significantly enriched. Finally, PLG, C8B and F2 were validated to distinguish between AMI patients and healthy people.

### 3.1. Exosome characterization of the plasma

**Figure 1.**
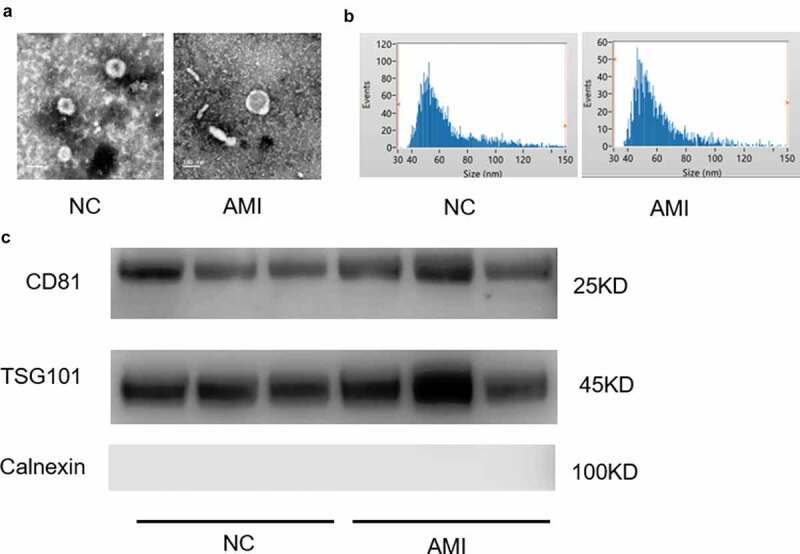
The characterization of exosomes from the plasm of AMI patients and healthy controls. (a) Representative electron microscopy images of exosomes secreted by plasm of AMI patients and healthy controls. (b) The size of exosomes from the plasm of AMI patients and healthy controls. (c) Detection of exosome-associated proteins (including CD81, TSG101, and Calnexin) by Western blot analysis.

Exosomes were isolated from the plasma of AMI and healthy people. The representative ECG images of the study groups were shown in Figure S1. The morphology and size of the exosomes were visualized with transmission electron microscopy (TEM) (Figure 1(a)). Exosome size and concentration can be identified by using nanoparticle tracking analysis (NTA), showing a modal hydrodynamic size of 40–100 nm for exosomes (Figure 1(b)). Characterization of exosomes from different human plasma isolates confirmed that AMI group and control group exosomes that were similar in size and quantity produced. Further, exosomal identity positive markers CD81, TSG101 and negative marker Calnexin were confirmed by Western blot for exosomes from plasma (Figure 1(c)).

### 3.2. Proteome profile and DEPs in AMI

**Figure 2.**
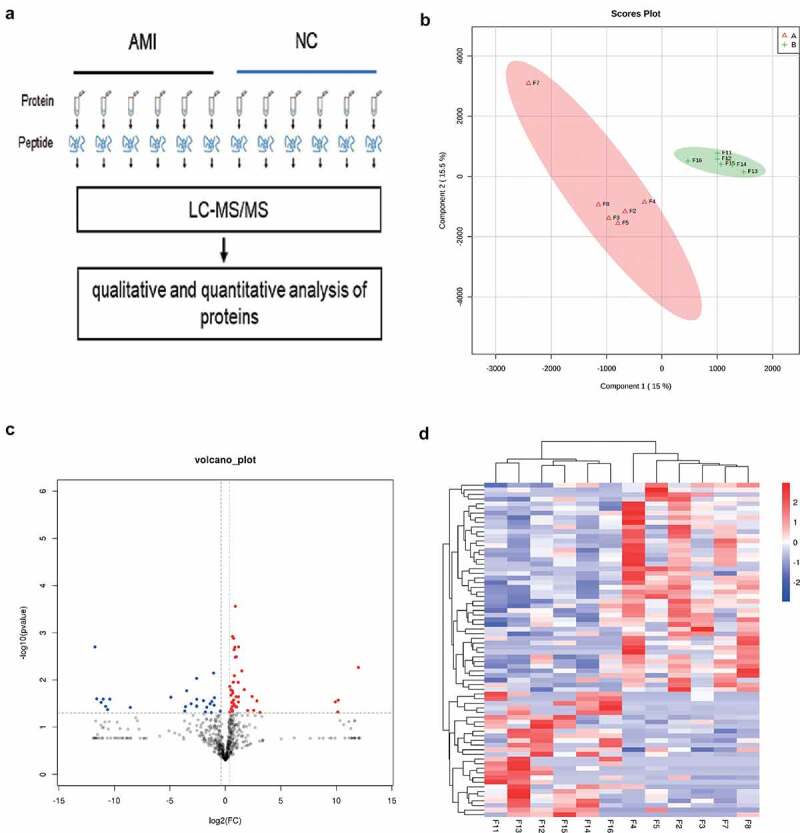
The label free quantitative proteomic landscape of the AMI patients and healthy controls. (a) The workflow of the label free proteomics in the study. (b) Principal component analysis illustrating moderate clustering of AMI patients and healthy controls. (c) Volcano plot of the differentially expressed proteins. Proteins significantly elevated in AMI patients or healthy controls are colored in red and blue, respectively. (d) Heatmap of the 72 significantly dysregulated proteins between AMI patients and healthy controls groups. Light blue represents the down‐regulated protein and red indicates the up‐regulated protein in the AMI patients group.

To identify the candidate biomarkers of AMI, we performed a label-free proteome screening experiment. In the present study, 12 samples, including 6 AMI and 6 healthy controls were performed by label free proteome analysis (Figure 2(a)). Unsupervised hierarchical clustering, along with PCA, was employed to compare the filtered list containing 789 proteins between AMI and 6 healthy controls. Most AMI and healthy controls samples clustered independently based on PCA (Figure 2(b)). Furthermore, 72 proteins were significantly changed (fold change > 1.3, P-value <0.05), of which, 27 and 45 proteins were downregulated and upregulated in the AMI group, respectively (Figures 2(c) and (d)).

### 3.3. GO enrichment analysis

**Figure 3.**
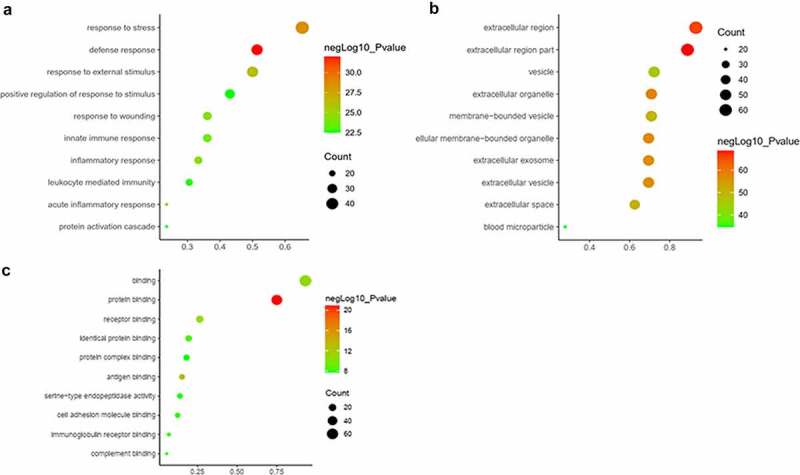
GO analysis of the differentially expressed proteins between the AMI patients and healthy controls. (a) Gene Ontology analysis conducted for the differentially expressed proteins in terms of the top 10 ranking biological process. (b) Gene Ontology analysis carried out for the differentially expressed proteins in terms of the top 10 ranking cellular component. (c) Gene Ontology analysis performed for the differentially expressed proteins in terms of the top 10 ranking molecular function.

All the DEPs were used to performed functional enrichment analysis. GO enrichment analysis was performed for these DEPs in three levels, including biological process, cellular component and molecular function. The top 10 biological processes were shown in Figure 3(a), including response to stress, defense response, response to external stimulus, positive regulation of response to stimulus, response to wounding, innate immune response, acute inflammatory response, inflammatory response, leukocyte mediated immunity and protein activation cascade. Obviously, these biological processes are mainly related to stimulus, immune and inflammatory responses. In terms of cellular component, majority of these DEPs are located in extracellular region, such as extracellular organelle, extracellular membrane-bounded organelle, extracellular exosome, extracellular vesicle, membrane-bounded vesicle and blood microparticle. The location information was matched with our study object, exosome (Figure 3(b)). Otherwise, most molecular functions of DEPs were binding, including protein binding, antigen binding, receptor binding, identical protein binding, immunoglobulin receptor binding, cell adhesion molecule binding, complement binding and serine-type endopeptidase activity (Figure 3(c)).

### 3.4. Pathway enrichment analysis

**Figure 4.**
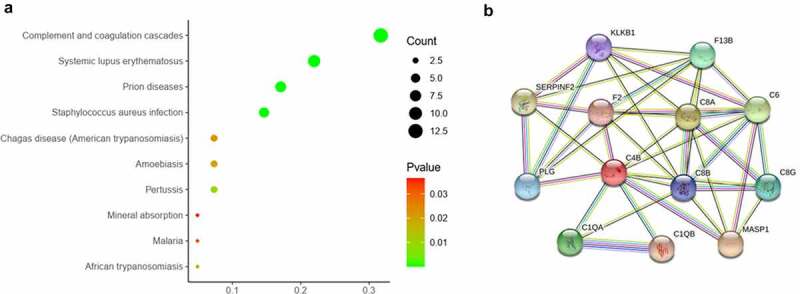
The pathway analysis of the differentially expressed proteins between the AMI patients and healthy controls. (a) The significantly enriched top 10 ranking KEGG pathway of the DEPs. (b) The PPI network analysis of DEPs involved in Complement and coagulation cascades by STRING database.

The pathway enrichment analysis was based on KEGG database. The most enriched top 10 KEGG pathways of the DEPs are shown in Figure 4(a). The most significant enriched term was Complement and coagulation cascades (p = 2.13e-16). There were 13 DEPs were enriched in this pathway, in which most DEPs were upregulated (Table 1). The protein-protein interaction of the 13 proteins were shown in Figure 4(b).

**Table 1.** The enriched top 10 KEGG pathway

Pathway Name	Pvalue	Genes
Complement and coagulation cascades	2.13E-16	C8B; C6; PLG; C8G; C8A; C4B; F2; KLKB1; SERPINF2; F13B; C1QB; C1QA; MASP1
Prion diseases	7.79E-10	C8B; C6; C8G; C8A; C1QB; C1QA; NCAM1
Systemic lupus erythematosus	5.84E-08	C8B; C6; C8G; C8A; C4B; C1QB; C1QA; HIST1H2BK; H2AFX
Staphylococcus aureus infection	0.000000767	ICAM; PLG; C4B; C1QB; C1QA; MASP1
Pertussis	0.0092	C4B; C1QB; C1QA
African trypanosomiasis	0.0174	ICAM; APOL1
Amoebiasis	0.0199	C8B; C8G; C8A
Chagas disease (American trypanosomiasis)	0.0221	C1QB; C1QA; CALR
Malaria	0.0326	ICAM1; SDC1
Mineral absorption	0.0364	TF; FTL

### 3.5. GSEA confirmed upregulation of complement and coagulation cascades in AMI

**Figure 5.**
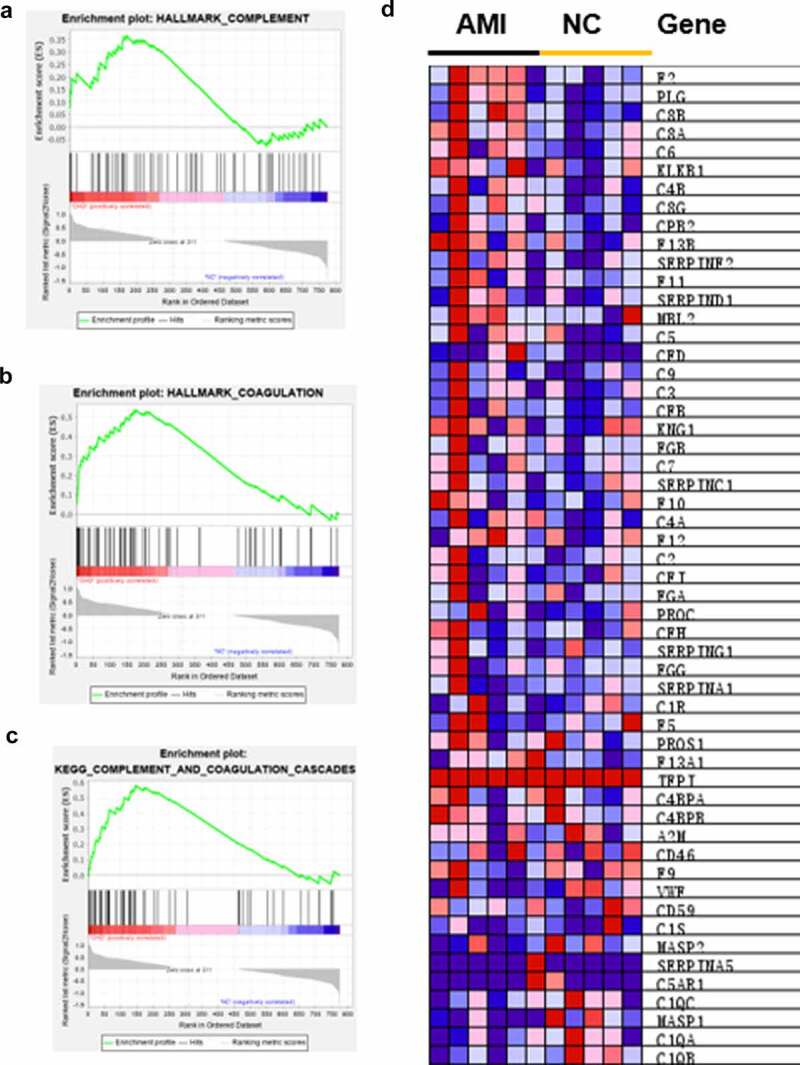
GSEA analysis of the whole quantified proteins between the AMI patients and healthy controls groups. (a-b) The hallmark genes of Complement and Coagulation were enriched by GSEA, respectively. *P < 0.05. (c) GSEA comparing for enrichment of the Complement and coagulation cascades pathway. *P < 0.05. (d) Heatmap of core enrichment genes in the gene set Complement and coagulation cascades.

Although the identification of specific DEPs in individualized patients is critical for possible biomarkers, it is equally important to elucidate the affected protein pathways in order to approximate the pathologic mechanisms. According to strict filtering criteria for protein modulation, the possibility of producing an insignificant result on a single member of these pathways is not consider [20]. Thus, all identified proteins were conducted by GSEA. The hallmark gene sets and KEGG gene sets were selected to perform the GSEA. We found that complement (Figure 5(a)) and coagulation (Figure 5(b)) were both upregulated in AMI by hallmark gene sets. Also, complement and coagulation cascades was upregulated by KEGG gene sets through GSEA (Figure 5(c)). The core enrichment genes of complement and coagulation cascades are shown in Figure 5(d).

### 3.6. Validation of PLG, C8B and F2 and ROC analysis

**Figure 6.**
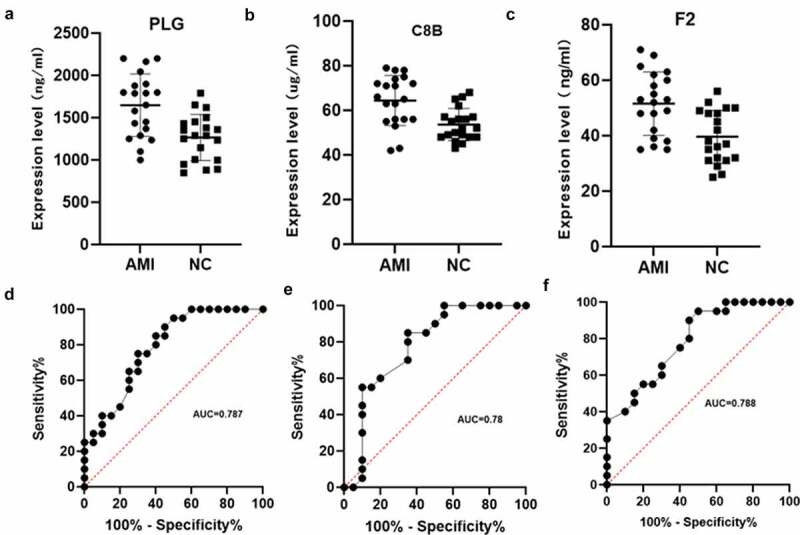
Verification using enzyme-linked immunosorbent assay of PLG, C8B and F2 in individual AMI patients (n = 20) and healthy controls (n = 20). Plasm levels of PLG(a), C8B (b) and F2(c) were significantly higher in AMI patients compared with control group (*P < 0.05). (d-f) Receiver operating characteristic curves of PLG, C8B and F2 between AMI patients and healthy controls.

Combined literature research and our results, PLG (Plasminogen), C8B (Complement C8 Beta Chain) and F2 (Coagulation Factor II, Thrombin) were selected from complement and coagulation cascades pathway as candidate factors between the AMI group and the healthy control group. Next, we verified another 20 AMI patients plasma and 20 healthy human plasma by ELISA assay. The results showed that PLG, C8B and F2 were significantly upregulated in AMI patient plasma (P < 0.05, Figure 6(a-c)). Subsequently, ROC curve analysis was used to validate the values of PLG, C8B and F2. The results are shown in Figure 6(d-f). The AUC of PLG, C8B and F2 were 0.787 (95% CI 0.647–0.927), 0.780 (95% CI 0.629–0.930), 0.788 (95% CI 0.651–0.926), respectively, which showed the mediator-differentiating effect of these proteins. This may suggest that these three proteins can distinguish between AMI patients and healthy people. Then, the correlation between clinical characteristics and the expression of the three proteins were performed. The results showed that the expression of PLG, C8B and F2 was significantly positively correlated with neutrophil % and significantly negatively correlated with lymphocyte % (Figure S2).

## 4. Discussion

Quantitative methods including ultra-sensitive mass spectrometry are undoubtedly one of the most widely used biomarker discovery methods in recent years [21–24]. Label-free quantitative proteomics were widely used for the discovery of potential biomarkers in cardiovascular disease and other diseases [25–27]. In this study, label free quantitative proteomics and validation were used to identify PLG, C8B and F2 as three potential biomarkers for AMI diagnosis.

These potential biomarkers may be useful for the diagnosis of early AMI [28]. According to our analyses, a total of 72 plasma exosome protein expression levels were significantly changed in the AMI patients, of which 45 proteins upregulated. By KEGG pathway enrichment and GSEA analyses, we found ‘complement and coagulation cascades’ pathway was activated. Subsequently, ELISA assays were used to verify the DEPs of this pathway. We validate the candidate proteins by another larger samples, and finally proved that the three proteins PLG, C8B and F2 were significantly upregulated in AMI patients.

Plasminogen (PLG) protein circulates in plasma as an inactive proenzyme [29]. It is converted into an active protease plasminogen by several plasminogen activators such as urokinase plasminogen activator (uPA), kallikrein, hageman factor and tissue plasminogen activator (tPA) [30]. As a regulator of the natural immune system, PLG can promote the phagocytosis of phagocytes [31]. The upregulation of PLG may cause an immunity response in the body [32] and the plasminogen system is important for tissue remodeling and inflammation. PLG has been shown to have a profound effect on cardiovascular disease and inflammatory response, which is the cause of these cardiovascular diseases [33]. Also, PLG may affect the progression of cardiovascular disease by degrading matrix proteins [33]. In our study, PLG was significantly upregulated in the plasm exosome of AMI. This may indicated that PLG funcrions in AMI, but the specific effect needs further experimental confirmation.

C8B is a subunit of the complement component 8 (C8) protein. C8 is a component of the membrane attack complex that mediates cell lysis and initiates membrane penetration of the complex [34]. It plays a key role in the formation of membrane attack complex (MAC), which is an important antimicrobial immune effect [35]. Studies showed that sC5b-9, C3bc, C4bc and C3bBbP were elevated in acute heart failure patients [36]. In addition, plasma C3D in AMI patients is higher than that in normal subjects, which may be a biomarker of AMI inflammation and tissue damage [37]. In our study, the complement protein C8B was also upregulated. This indicates that it may have a similar indicator role as C3D in AMI

F2 (thrombin) is a key protein in the coagulation cascade. The activated thrombin enzyme plays an important role in thrombosis and hemostasis by stimulating platelet aggregation, converting fibrinogen to fibrin during blood clot formation, and activating additional coagulation factors [38]. It also function in tissue repair, cell proliferation, angiogenesis and maintaining vascular integrity [39]. Otherwise, thrombin is a pro-coagulant and pro-inflammatory serine protease that contributes to the pathology of atherosclerosis by enhancing the expression of cell adhesion molecules [40]. Similarly, thrombin was found to upregulate in AMI group in our study. It means that AMI patients have increased cell adhesion to their blood.

## 5. Conclusion

Taken together, using label free proteomics strategy, integrated bioinformatics and validation, PLG, C8B and F2 were considered to be three potential biomarkers for AMI diagnosis. However, the role of the validated proteins in AMI is still poorly understood and needed further functional studies. Furthermore, more cases will need to be investigated in the future to verify the conclusions in the study.

## Supplementary Material

Supplemental Material

## Highlights

- Seventy-two differentially expressed proteins were identified in AMI patients’ plasma exosomes.
- Complement and coagulation cascades pathway was activated in AMI group.
- PLG, C8B and F2 may be potential biomarkers for AMI diagnosis.

## Data accessibility

All the data will be provided by reasonable request from the corresponding author.
