# Supplementary figures and images for: Quantitative proteomics reveal three potential biomarkers for risk assessment of acute myocardial infarction
Source: Bioengineered. 2022 Feb 14;13(3):4939–50. doi: 10.1080/21655979.2022.2037365 (PMC8973584; doi:10.1080/21655979.2022.2037365)

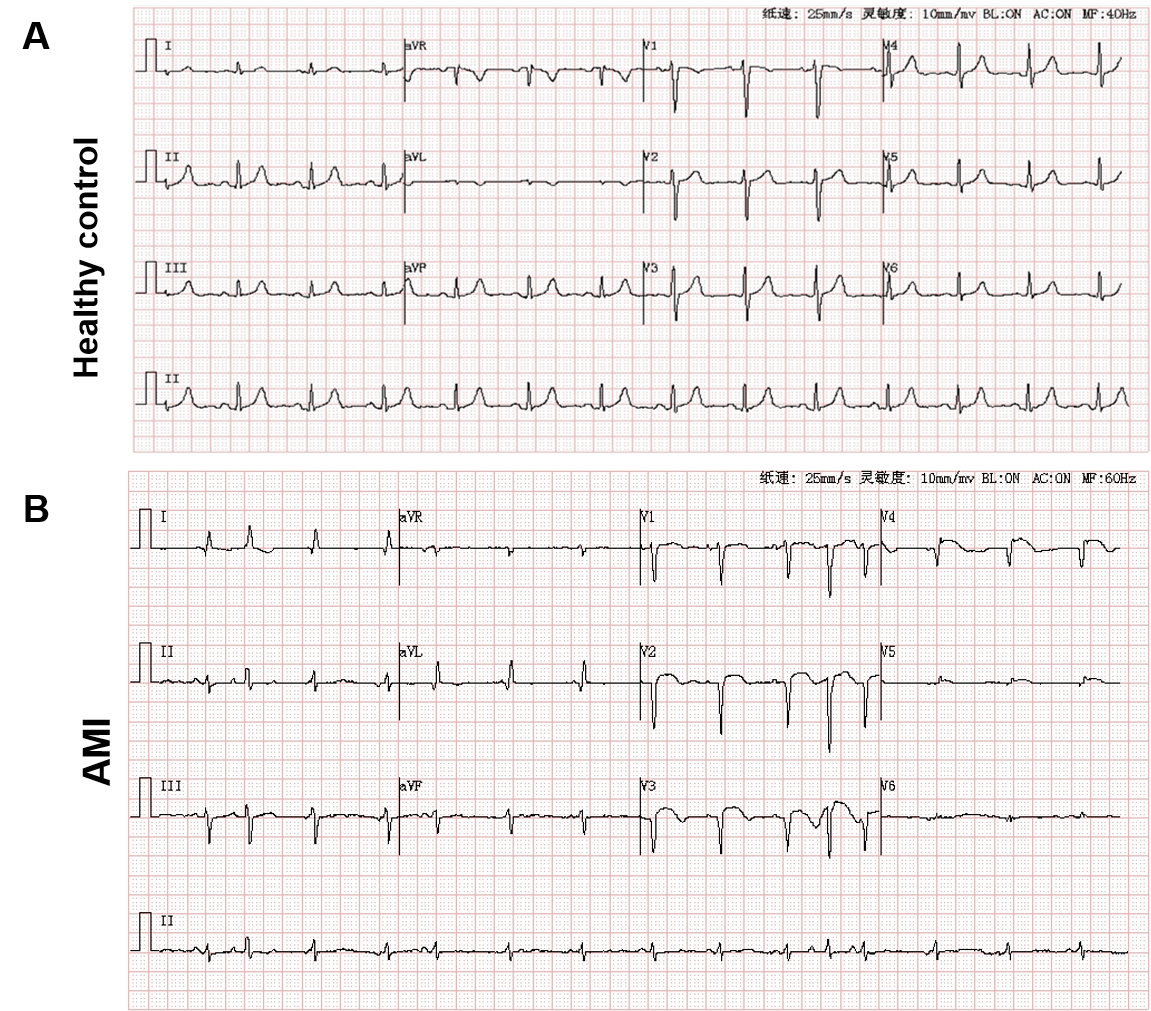

Supplement: Supplemental Material [file KBIE_A_2037365_SM1739.zip › supplementary/Figure S1.tif]

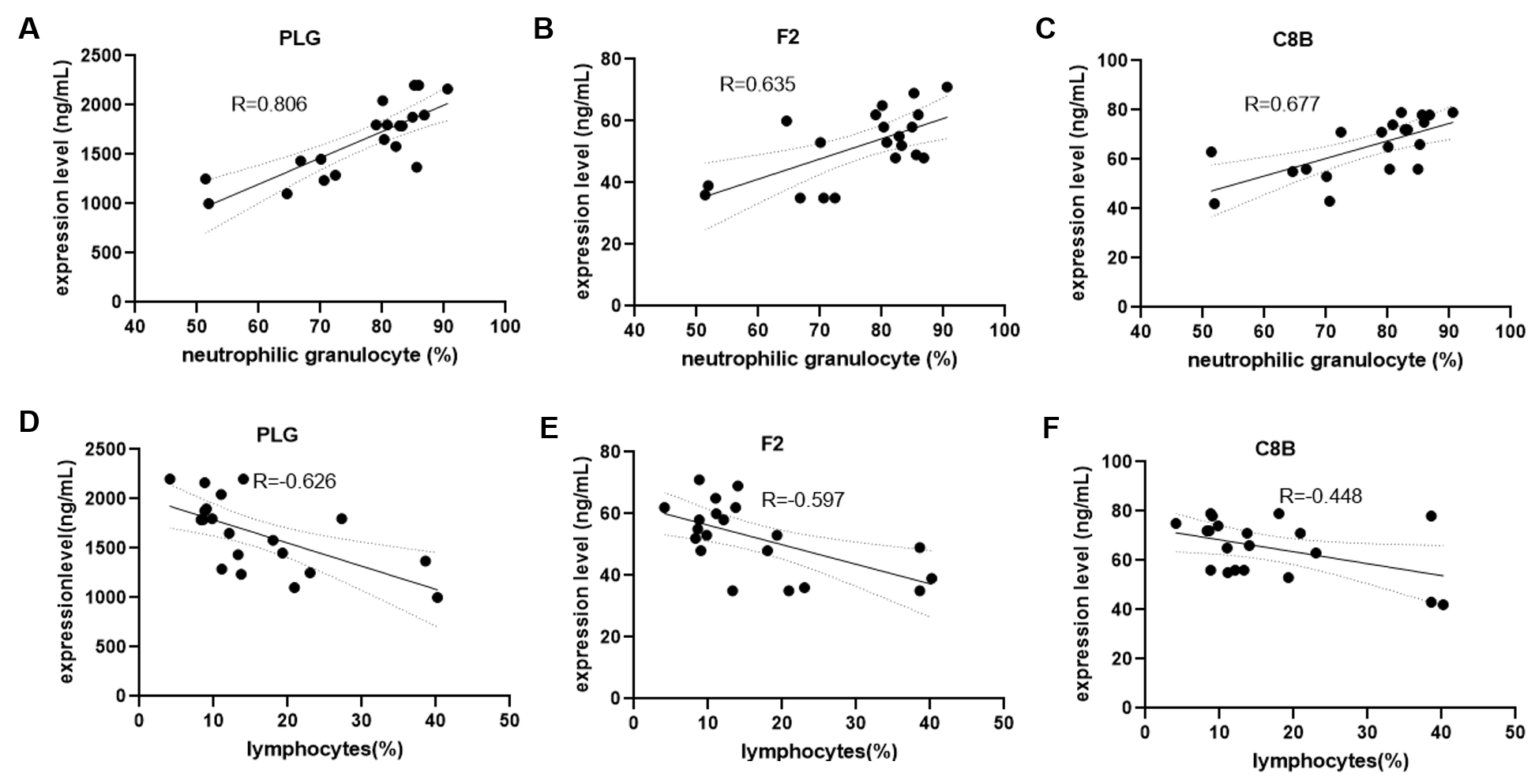

Supplement: Supplemental Material [file KBIE_A_2037365_SM1739.zip › supplementary/Figure S2.tif]
